# Policy initiation and political levers in health policy: lessons from Ghana’s health insurance

**DOI:** 10.1186/1471-2458-12-S1-S10

**Published:** 2012-06-22

**Authors:** Anthony Seddoh, Samuel Akortey Akor

**Affiliations:** 1 Centre for Health and Social Services, PMB 52 Ministries, Accra, Ghana; 2 Mount Crest University College, Read-wide House, Accra, Ghana

## Abstract

**Background:**

Understanding the health policy formulation process over the years has focused on the content of policy to the neglect of context. This had led to several policy initiatives having a still birth or ineffective policy choices with sub-optimal outcomes when implemented. Sometimes, the difficulty has been finding congruence between different values and interests of the various stakeholders. How can policy initiators leverage the various subtle mechanisms that various players draw on to leverage their interests during policy formulation. This paper attempts to conceptualise these levers of policy formulation to enhance an understanding of this field of work based on lived experience.

**Methodology:**

This is a qualitative participant observation case study based on retrospective recollection of the policy process and political levers involved in developing the Ghana National Health Insurance Scheme. The study uses a four-concept framework which is agenda setting, symbols manipulation, constituency preservation and coalition building to capture the various issues, negotiations and nuanced approaches used in arriving at desired outcomes.

**Results:**

Technical experts, civil society, academicians and politicians all had significant influence on setting the health insurance agenda. Each of these various stakeholders carefully engaged in ways that preserved their constituency interests through explicit manoeuvres and subtle engagements. Where proposals lend themselves to various interpretations, stakeholders were quick to latch on the contentious issues to preserve their constituency and will manipulate the symbols that arise from the proposals to their advantage. Where interests are contested and the price of losing out will leave government worse off which will favour its political opponent, it will push for divergent interests outside parliamentary politics through intense negotiations to build coalitions so a particular policy may pass.

**Conclusions:**

This paper has examined the policy environment and the political leverages in retrospect at arriving at Ghana’s health insurance policy and design. New perspectives have been brought to the dynamics of the interactions of the 3 streams of problem, policy and politics. It provides lessons which suggest that in understanding the policy process, it is important that actors engage with the content as well as the context to understand viewpoints that may be expressed by interest groups. This will empower policy proponents to achieve easier results and limit the frustrations associated with the policy process. There are no straight and determined pathways for achieving outcomes so appreciating the evidence and basis for design, negotiation process and building coalitions along the way are skills to be mastered.

## Background

It is recognised that implementing universal social health protection may be one of the critical ways of achieving the MDGs and universal health coverage. Providing access to health care, including equitable health insurance mechanisms, are important contributions to eradicating poverty and vulnerability. Such mechanisms address poverty and vulnerability on several levels [1]. The introduction of Social Health Insurance into low to lower middle income countries has been slow in coming. According to an ILO Social Security Policy briefing paper on Social Health Protection [2], for many years it was generally believed that extending social health protection into developing countries was premature because such countries are economically immature to deal with the political and financial burden associated with social security. The objectives of social health protection policy relate to the entire population and in developing countries the financial compromises required are excessively sub-optimal. The difficulty is that objectives are not always congruent for different players with different value interests. This has led to some authors concluding that public sector policy is inherently political. Social health insurance policies adoption is no exception.

There are several publications on the technical aspects of health insurance in countries and its effects on the financing environment. Various systems of social health insurance and their premium payment mechanisms exist. Premiums may be paid at household or individual level. In his view, using the household as a basis for payment means that households pay a fixed amount irrespective of seize and this helps to combat adverse selection. Lewis [3] examined the technical factors in design and its effect on institutional management outcomes and observed that the institutions developed can lead to excessive bureaucracy, cost escalation and difficulties in premium management. Ellis and Miller [4] in their analysis suggest that the introduction of social health insurance in countries shifted governments from the use of budgets as the main system of paying for services to either a fee for service model (FFS) or a patient-based payment method (PBP) such as diagnostic-related groups (DRGs). Shifting from one to adopt the other can lead to complications in health financing and insurance management and required technical expertise that may not always be available to developing countries.

There is very limited literature available on the political factors influencing choice of design. It is only Agyepong and Adjei [5] who examined the power dimension that comes to play between political and technical experts during the introduction of health insurance in Ghana. It noted that the power of the politically powerful was used to sieve out undesirable inputs from technical experts which do not add to their intended outcome. The authors observed that “… the stronger position of political actors to control and direct the policy process, combined with some political sense of insecurity” ”(p 158) was what determined the policy outcome. Technical experts and other stakeholder roles were expressed as passive, placid or something that can be placated. They were critical of the seeming powerful political elite in selective engagement and interest mongering with an almost abrasive militancy. It however did not explain why particular policy decisions were adopted to arrive at the existing design. There is also no publication that unpacked and conceptualised the political leverages that are drawn on to provide lessons for agencies and institutions seeking to introduce a major public sector reform such as health insurance.

The focus of this paper is to partially fill this gap by drawing on a framework of policy analysis to bring attention to the political levers in Ghana’s health insurance policy development. It adds a new dimension to the policy and political leverages that influenced realised policy and contributes to literature both in terms of the framework of analysis and the political context. The idea is to draw out lessons that may be applicable widely in the health policy analysis and development process.

## Framework for analysis

There are challenges in doing health policy analysis because of the complexity involved in the different definitions associated with it [6]. There is conceptual debate over whether policies emerge or are planned, whether the issues are observable objectively and whether these manifest in a consistent pattern. As Walt and others [7] noted, “there are also many other conceptual challenges … capturing and measuring the levels of resources, values, beliefs and power of diverse actors is difficult’ (p 310). A number of authors have recommended that attempts to understand why a particular policy was adopted should revolve around factors related to agenda setting, allocating resources, distribution power and deciding whose needs come first [8-10].

In the various publications where power resources are used to explain policy adopted the broader concerns are usually expressed as affecting interests preserved by or imposed on specific groups in societies by powerful elites. Gringle and Thomas’[8] work in developing countries is one of the most quoted paper in understanding the role of political actors in influencing policy. The authors suggested that government and public officers constitute into an elite group and have a strong influence on agenda setting and the nature of policy adopted. The elite actors work together to protect their interest from the elegy and interest of actors not within the circle of official positions. This act of preservation is to protect ideological positions and retain control over the agenda and power resources. The agenda setting circumstance has a particular peculiarity which generally arises out of the environment context, particularly in circumstances of crises and reform.

Thomas and Gilson [11] others based on experiences from South Africa and Zambia observed strong influence of in-country political factors and actors over which health care financing policies were implemented, which should not and what the details of policy design should be. Prominent among these are ministers of health and finance, parliamentarians, academic analysts and trade unions. They noted that technical experts and analysts, working either inside or outside government, had varying and often limited influence on the type of policy to be adopted. In part, this reflected the limits in the technical understanding and capacity of these experts as well as weaknesses in the way they were used in policy development.

The main contribution of the authors cited to the discourse in public policy lies in appreciating the leverage of those in authority mostly the elected or the executive within the public sector and their ability to sift out inputs that are incongruent with their aspirations to arrive at preferred agenda and policy outcomes. They reiterate Dye’s [12] idea of the political powerful where in democracies such as the United States; public policy is made from the top down, not from the bottom up. It is important to note that all the authors subtly recognise the need for technicians to learn the politics of reform but did not explore the how. However, there is very limited analysis on the cyclical reflection of other groups’ negotiation power in arriving at outcomes. Between the various authors, the multiple associations and networks which draw on different levers of power resources based on discourse and negotiations that influence policy are filtered out in favour of power produced through class relations. Fundamentally, the business of governance does not support this strict dichotomy between politicians and technicians.

To assume that power in determining policy rests only with the political elite and in a context of givers and takers will need further interrogation if we are not to be caught up in a single dimension framework. Gramci’s [13] idea of counter-hegemonic manoeuvres drawing on multiple positions to influence the outcome of decisions such provides anti-thesis. It brings in a dimension of social construct that serves to legitimate other social structures in influencing policy. Public policy is a result of on-going negotiation between different actors who have almost an equal influence on direction and outcome. These actors include trade unions and political parties who gain concessions using ‘manufacture consent’ and legitimacy [14].

Kingdon’s [15] multiple-streams theory also provides a direction for multiple analyses beyond the power of the elite. Here the process of public policy formulation is randomly engaged where problems, policies and politics interact in independent streams. The various streams flow into each other as various constituents settle on issues that provide common opportunities and agreements. Here technical experts engage strongly sometimes as a moral imperative, which sometimes may result in a value conflict and should be seen as a sign of healthy administrative practice.

**Figure 1:**
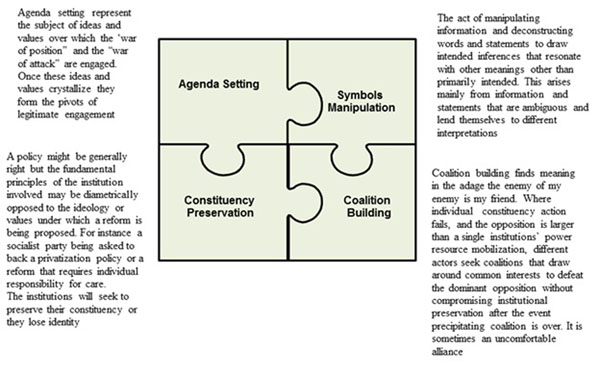
Four political levers of policy engagement

Clearly there is considerable strength in looking at the influence of the political elite in setting the policy agenda and arriving at a specific policy outcome. Their actions of preservation of interest to protect ideological positions and retain control over the agenda and power resources are significant in any analysis. However, to understand the entire process and elements, it may be important not to confine analysis to the political elite. The social constructs that recognise the influence of other players such as civil society and the policy stream which include organisational, symbolic and scientific evidence has to be equally considered. These various constituents and elements when woven together provide a complete nexus of evidence on the key factors to be considered in the policy formulation process. Based on appreciation of these various factors, it is possible for our purpose in this paper to regroup the various elements into four thematic areas: agenda setting, symbols manipulation, constituency preservation and coalition building. We refer to these as the policy levers drawn on by various actors to negotiate a policy outcome. The levers are elaborated in figure 1 below. The paper uses these levers to explain the experience of Ghana in developing its National Health Insurance policy, and draws lessons for actors preparing to introduce major public policy reforms. The four theme levers were adopted because they capture the most recurring observations and lived experiences in the policy development process as we saw it.

## Methodology

This is a qualitative study based on participants’ observation. Participant observation was used to capture lived experiences by the authors as a key member of the entire process while observing the events unfold and having an understanding of the decision making process. It helps to answer questions on explanatory values of alternative action. As noted by Agyepong and Adjei [5], “the in-depth personal engagement of a participant observer yields rich and ‘thick’ descriptive material and insights” (p 153) but it is also its weakness. The participant’s view of the process may be clouded by their biases and sentiments and their influence on the processes may not be objectively assessed. This is substantiated by Walt and others [7] when they noted that “one of the issues facing health policy analysts is how they are viewed or situated as researchers” (p314). One cannot objectively observe one’s self and how the participant’s behavior affected outcomes. The participants’ views, preferences and opinions can also form a lens and filter out what is undesirable and incongruent with the personal beliefs, norms and values. To minimize this level of bias, the authors avoided were explicit where their judgment is inferred and discussed issues to show how they were used to arrive at outcomes. The author’s involvement with the process of health insurance development in Ghana is explained in endnote.

## Factors leading to the establishment of health insurance

Ghana’s 2010 population was estimated at 24.3 million. Overall poverty rate had declined substantively over the past two decades from 57.7% in 1991/92 to 28.5% in 2005/2006. The proportion of the population living below the extreme poverty line also declined from 36.5% to 18.2% over the same period. Ghana benefitted from the Highly Indebted Poor Countries (HIPC) initiative. The health sector was allocated approximately 20% and 18% of poverty reduction related funds in 2007 and 2008 respectively. An important aspect of Ghana’s population structure relates to ‘population momentum’. Ghana’s dependency ratio currently is .72 dependents per productive member. About 67% of the population is in the informal sector.

The economy remains predominately agrarian though it is suggested that the service sector may probably be contributing more to GDP. From 2001 growth began to accelerate and reached a high of 7.3% in 2007, which is the second highest growth rate in the past three decades after the 8.6% recorded in 1984. In the wake of the global financial crisis and economic decline in 2007, the real GDP growth rate declined to 4.7% in 2009. Value Added Tax was introduced in 1998 and partial implementation of universal Tax Identification Numbers was introduced in 1998. Price subsidies on agricultural inputs were removed and independent utilities regulatory commission was established in 1998.

The current estimated life expectancy rate is 57 years; Total Fertility Rate is 4.0 in 2008 compared with 4.4 in 2006. Use of modern contraceptive is about 17% in 2009. The crude death rate is estimated at 8.93 deaths/1,000 population as of July 2010. Maternal mortality ratio (MMR) between 1990 and 2007 had reduced from 740 per 100,000 live births to 451 per 100,000 live births [16]. At this rate, however, Ghana may not attain its MDG 5 target of 185 per 100,000 live births. It is estimated that the MMR will be 340 per 100,000 in 2015. The Ghana Demographic and Health Survey [17] showed a reduction in the under-five mortality rate from 111 per 1000 live births in 2003 to 80 per 1000 live births. Infant mortality rate was 50 per 1000 live births in 2008 compared to 64 in 2003 with neonatal mortality rate decreasing from 43 per 1000 live births in 2008. From the information, the most significant contribution to under-five mortality is deaths occurring within the first 28 days. Generally Ghana is considered on course to attaining the child malnutrition target under MDG 1 ahead of 2015. However, according to the 2008 GDHS, 28% of Ghanaian children were stunted, with 10% being severely stunted. The extent of wasting has worsened within the last five years. HIV prevalence among the adult population as at the end of 2009 stood at 1.9%.

• *Fee for service as a catalyst for health insurance*

Health financing in Ghana at post-independence was based on a “free” Health Services for All concept (tax-based, non-earmarked), which guaranteed universal access. In this regard, financial barrier to healthcare access was avoided. The sustainability of this approach to health financing was undermined by the persistent decline of the economy in the late 1960s. The Health sector competed with other sectors, especially the priority sectors for budgetary allocation from the consolidated fund. Thus, it suffered from inadequate funding during the period. This triggered the search for alternative or complementary health financing [18].

The Ghana health insurance policy agenda emerged from different factors and sources. The first proposal for a national health insurance scheme was made in 1970 as part of a comprehensive plan to introduce cost recovery and protect the poor. Though fee-for-service was introduced in 1971 nothing was done for insurance. The Fee-for-service (FFS) was implemented in a limited form as token fees in the absence of a Legislative Instrument. It was not until 1985 when the Legislative Instrument, LI 1313 was promulgated. Exemptions were granted for selected public health diseases including leprosy and yaws, hospital accommodation and antenatal and post-natal care. No provision was made for the poor, indigent and emergency care.

User fee became known in operation in Ghana as the ‘cash and carry’ system, reflecting its ‘supermarket approach’ and resulted in a decline in service utilisation. In the late 1990s, the attendant effects of FFS on utilisation became a source of concern for Government. The per capita OPD attendance had consistently dropped from 1.9 in 1970 to 0.3 in 2000. A study in 1999 [19] found that 84% of patients eligible for exemptions were not exempted. Another review [20] also noted that there was no clear guidance on how to identify the various persons to be exempted. In particular knowledge about how exemptions should be applied varied widely among various providers. Other difficulties included whether the exemption of a disease also included the exemption of the administrative cost associated with it before diagnosis and what happens when a condition manifest as a complication of other ailments.

Just about 44% of those who were ill had consulted a medical practitioner mainly because of the barriers that fee for service presents [21] at the time. Between 1995 and 2003 clinical attendance has fallen by 25% while the average revenue had increased. The drive for more revenue also led to over prescription of drugs beyond the acceptable prescription protocol for ailments such as malaria [22] and; rates being quoted as claims for reimbursement for drugs used varied widely for the same conditions and illnesses between facilities and regions [23]. This occurred because of the absence of a standard and reliable price list for conditions and commodities. As a result it was difficult to properly track the accuracy of what was being claimed. The general average reimbursement rate in 2003 and 2004 was 49% and 69% respectively.

• *Experience from early attempts at social health insurance*

Between 1989 and 1993 the Catholic Diocese of Sunyani initiated a health facility-based health insurance at St Theresa’s Hospital, Nkoranza in response to the negative effects of the FFS on service utilization. The decision to undertake this venture was inspired by the example of Bwamanda Hospital Health Insurance Scheme in the former Zaire and spearheaded by Dr. Ineke Bossman, then Administrator and District Medical Officer of Health (DMOH) in charge of Nkoranza at the time [24]. Although the Nkoranza scheme achieved significant enrollment of membership, it had its shortfalls among which were exclusion of the poorest of the poor, absence of community ownership and a precarious financial position to ensure sustainability.

The challenges notwithstanding, the Nkoranza experience inspired other initiatives. The Society of Private Medical Practitioners, Ghana in 1993 also established the first private mutual health insurance; Nationwide Mutual Medical Insurance Scheme in the country. By 1998 the scheme had collapsed as a result of delayed reimbursement due to manual processing leading to under-servicing on clients by providers, fraudulent claims and inadequate premium. New district schemes also emerged sponsored by some development partners at Damango, Drobo, Duayaw Nkwanta, Berekum and Dangme West districts. There was also no legal framework to regulate the business. However, the community having experienced the benefit of an insurance type prepayment arrangement were willing to sustain social insurance type mechanisms to finance their health care. Asenso-Okyere and others [25] had examined the willingness to pay for health insurance using Contingent Valuation Methods. The studies concluded that pre-payment schemes could curtail self-medication and delays in seeking care. There was a high degree of acceptance of health insurance with up to 63.6% willing to pay a premium of about US$3.03 per month.

## Milestones in establishing the scheme

The Ministry of Health, in 1997, under the National Democratic Congress led government established a task force and a Directorate for Health Insurance with the responsibility to facilitate the National Health Insurance Scheme. Preparatory efforts were made by commissioning an actuarial study and the Ghana Health Company, a subsidiary of SSNIT, established to initiate a health insurance scheme for the formal sector. It further went ahead to create a regional secretariat in Koforidua, Eastern Region. However, not much was done in actualising the dream at the time of handing over to the New Patriotic Party (NPP) led Government in 2001. The Division was abolished and the company was liquidated. The regional secretariat however remained but with no clear responsibilities.

The NPP manifesto gave an indication of abolishing “Cash and Carry” and replaced by a national health insurance scheme when elected into office in 2001. This was actualised by linking it to the main policy strategy of Government to reduce poverty contained in the Ghana Poverty Reduction Strategy I document and the Health Goal of Health for All. Thus, the focus was to ensure that the poor and vulnerable in society have access to healthcare. A Multi-Agency Technical Committee led by the Ministry of Health was set-up in 2001 comprising key stakeholders to formulate a policy and design the structure and form of the National Health Insurance Scheme. In drafting the policy, the committee had to conduct a critical analysis of eight (8) alternative scheme designs including the Kenyan and Tanzanian models and finally selected the decentralised approach; the District Mutual Health Insurance approach which was a cross between the Dangbe West District and Atiwa model. Whilst working at the policy, the Ministry initiated a pilot of the design at Ejisu-Juaben District in Ashanti region and subsequently at Kpeshie in the Greater Accra region. The idea was to test how the design will play out in both rural and urban settings. Being satisfied with the initial outcome, the Ministry decided to increase the number of schemes from about 10 to 45 so as to get a regional spread and learning experience in establishing the systems without access to benefits as yet. This was before the final policy was completed and the law passed. There was political commitment and clarity of direction from the political leadership and; funds were available from the Highly Indebted Poor Countries relief fund. Health insurance was one of the performance measures signed into the Minister of Health’s letter of appointment by the President. But there were also detractions from those who preferred a civil service approach of policy approval before action. This led to a number of fall outs within the committee with some members walking out and others considering the movers as traitors or ruling government sympathisers. The motivation however was simply to get things rolling before the enthusiasm gets paralysed by excessive analysis and technical detail. On hind sight, this proved to be a good approach as the rancour that accompanied the policy and legal development process discussed later in this paper will have truncated the entire exercise.

By January, 2002, a draft policy had been formulated which was put before stakeholders across the country grouped in four zones and a national forum. Subsequently, there were presentations made to specific stakeholder group, such as the Ghana Employers Association, the National House of Chiefs and the Trade Union Congress. The final draft was tabled by the Minister of Health at cabinet in May, 2002. Apart from the scheme design, the policy proposed various sources of funding of the scheme including the use of SSNIT contributions and VAT. The policy focus was to achieve redistribution of wealth through cross-subsidisation and risk-equalisation and therefore, the strategy was to make the scheme universal for both the formal and informal sectors to make contributions. The technical committee made two presentations at cabinet. After the first presentation, a sub-committee of cabinet was constituted to carry out further analysis. The cabinet sub-committee requested the technical committee to provide it with detailed financial analysis to assist it in arriving at a decision.

It took cabinet about six month to decide on the financing model. The policy was approved in December, 2002 and an announcement was made through the 2003 budget submission to create a health fund for the health insurance scheme with a National Health Insurance Levy of 2.5% on consumption goods and hiving off 2.5% of the 17.5% contributions to the SSNIT fund representing contributions of the formal sector workers. The technical committee made a presentation on the draft bill to the Joint Parliamentary select committees on Health and Subsidiary Legislation. Ghana’s National Health Insurance Scheme was finally introduced in 2004 following the passage of the Act of Parliament, Act 650 of 2003 and Legislative Instrument 1809, 2004. . In 2005 the technical committee moved quickly to expand coverage to 125 districts to undertake preparatory activities to establish full blown district mutual health insurance schemes as soon the Act became operative.

## Political levers influencing policy and design

• *Agenda setting*

Publications in relation to failed fee-for-service policy implementation and experience with social health insurance schemes provided good material to convince those who were evidence inclined. These were translated into policy briefs and flyers in simple language to bring attention and catalyse discussion among key stakeholders. There was a deliberate and systematic effort by the Committee members to engage think tanks and civil society organisations such as the Institute of Economic Affairs, the Ghana Medical Association and academic institutions to interrogate and debate the merits of an insurance scheme. The Health Partners Summit held twice in a year also became a convenient platform for keeping the agenda on the table. Between 1999 and 2002 the effect of user fees and health insurance featured on every health summit and was captured in the aid memoire of four of the summits as the preferred policy.

However, to get the health insurance to become a national agenda required more than academic evidence and aide memoirs. It had to be translated into the realities of the population lived experiences to which the political decision makers and legislators can relate. The technical experts turned to the media for support. Primarily, civil servants started exposing the media to the challenges of the sector. One of the reported issues within clinical practice was what became known as ‘medical prisoners’. These were in-patients who have been cured of their ailment but are being detained by health facilities until their debts were redeemed by relatives or philanthropists. The uncomfortable effect of these publications led to politicians asking questions about what could be done to resolve the issues.

As ministers turn to technical experts for advice, they pointed to health insurance as a way out and ‘sneaked’ them into speeches of ministers as an issue on the table to ameliorate the situation and held several discussions on radio and television. However, the debate on what form insurance should take was not without its own complications. The debates at the various forums and in the media sometimes polarised the issues along political lines and the facts got misrepresented particularly when non-technical persons within the media attempted to translate technical words into vernacular or local dialects. Some of the words simply could not be translated. For instance, it was difficult to precisely translate premium into a single word. Attempts to use imagery and figurative sentences sometimes complicated understanding and lent it to manipulation. There were also so many interests and complications to be negotiated of which constituency preservation was key.

• *Constituency preservation*

The pluralism of pilot models caused public opinion on the models proposed by the National Democratic Congress (NDC) to be so diverse that major political parties started issuing statements on the implementation of the scheme in order to preserve their constituencies. Each political party defined what their positions were while attacking the position of the other.

The major political parties engaged in a battle of the hearts and minds of the electorate. Each party attempted to represent itself as having something distinct from the other but not always with clarity. It however did not matter in so long as a certain noise was generated which attacked the other parties positions as unacceptable propositions. It was not clear what the National Democratic Congress (NDC) government that started the current phase proposal’s position was and it is still very difficult to date to articulate what its policy is on health insurance. The NDC had no written document on their position on health financing and this made it difficult for its constituencies to align with a single vision. Its position was generally a cross between individual responsibility for health but without alienating itself from the core principles of social democracy. Providing government subvention in health financing was seen as providing that opportunity.

The lack of clarity in its position on the matter made it difficult for its followers and members of parliament to defend the party agenda on insurance and decimated its constituency. One of its deputy Ministers of Health in 2000 argued that a single national system as was being piloted was not feasibly and called for district schemes. This did not sit well with his colleagues in parliament. He was accused of selling out and forced to reword his presentation during questioning in parliament on the issue. The lack of focus led to the introduction of multiple pilot schemes with none being given priority attention for replication.

The New Patriotic Party (NPP) in their medium term priorities made clear its intent to phase out the cash and carry system and replace it with a more humane and effective system of financing health care. The party on assuming power in 2001 quickly sent out a clear sign of government intent on introducing insurance. As noted earlier one of the key performance measures in the letter appointing the Minister of Health to the position in October 2001 was the introduction of social health insurance. On December 19 2001, the Minister of Health announced the processes for drafting of a bill that would regulate the health insurance industry, create a national insurance fund and provide procedures for resolving conflicts. This allowed its followers to comment on issues being discussed by the public even if sometimes their arguments were incoherent. What clarity did for them was that they were able to rally support among party faithful, gave the voice to participate in debates and preserve their constituencies.

When government position is not clear, technical experts turn to preserve their own constituencies by ignoring government directives or engage with it in a lukewarm manner usually resulting in the failure of the policy. The relative clarity on the part of the NPP and its commitment to achieve the introduction of health insurance gave technical experts within the Ministry of Health a direction on what the government policy was without compromising the position of Ministry of Health officials as pursing a non-technical agenda. In our opinion, it is this lack of clarity on the part of the National Democratic Congress when it started its own schemes that contributed to its inertia.

• *Symbols manipulation*

Symbols have long been known to be manipulated to both positive and negative by various actors. This is because words and statements presented are not always ubiquitous in meaning particularly if they are ambiguous and can be reinterpreted and re-presented.

The policy paper submitted to cabinet contained a proposed design of health insurance and the possible financing sources. Three scenarios were presented for financing. The first was to raise funds based entirely on premiums as derived from the ILO actuarial analysis. This will have meant a minimum of the equivalent of US$360 per annum for each citizen if the entire population was to be covered for a suggested benefit package mainly outpatient services and referred cases. The second was to draw funds from three sources: government budget, payroll deductions of 15% from formal health workers and an additional contribution of the equivalent US$120 from the entire population irrespective of employment status per annum. The third is to drop the contributions entirely, replace payroll deduction with allocations from the Social Security and National Insurance Trust fund, source funds from government budget and raise the percentage on the ad-valorem tax (VAT) by about 3.5% and allocate that to the insurance fund.

The first two were rejected on grounds that at a per capita income of US$360 at the time and the level poverty these were not feasible propositions. On the third, the reaction from cabinet was that using ad valorem tax (VAT) though attractive posed its own political problems. VAT as a symbol will easily be manipulated by the opposition. The National Democratic Congress introduced VAT in 1998 when the NPP was in opposition. The NPP described the tax as regressive, in-human and insensitive to the plight of Ghanaians. Following very heated debates and heckling in parliament and casualties resulting from a demonstration dubbed ‘kumi preko’ meaning ‘better kill me now’ insinuating that it is better to die than live under a VAT regime, the NPP then in opposition parliamentarians walked out of the proceedings and refused to vote on the issue.

The NDC passed VAT into law because they formed the majority in parliament then. To mention an increase in VAT by any figure will be playing right into the hands of the NDC now in opposition. After a lengthy debate, it was agreed to call it a National Health Insurance Levy (NHIL). At Akosombo in 2003 where the Parliamentary Sub-committee on Health met to deliberate on the matter, there appeared to be bi-partisan consensus. When the policy was tabled in parliament under the draft bill, the NDC were quick to latch on the NPP ‘double standard’ and decried a tax increase that will translate into higher prices on goods and services. It argued that a tax that is collected by the VAT agency, on the same qualifying goods and services and administered under the same tax regime can be nothing but an increase in VAT.

The debates on the floor of parliament were uncompromisingly polarised, technically deficient and focused on government insincerity at opposing a previous policy on which it is building its fortunes. The images and accounts of mass demonstrations led by the NPP, the loss of lives, the embarrassment of withdrawing an earlier VAT bill in 1995 and reintroducing it three years later with a much lower rate and loss of revenue were too strong to ignore. The NDC recounted these vividly on the floor of the debate and raised strong sentiments among its sympathisers against the proposal.

Academicians joined in the debate and put out the mathematics to show that the increase of 2.5% is actually an increase of 20% in the tax regime. The production sector protested that the new levy will increase production cost and make them uncompetitive against imported finished products. The general population weighed in through mass media but the president and his constituents were resolute in their resolve. In almost a payback faction, the NDC walked out of parliament when it came to vote on the issue. The NPP being in majority voted to pass the bill.

• *Coalition building*

Enrolment and membership in a District Mutual Health Insurance Scheme is mandatory for all residents of Ghana except those working with the Ghana Armed Forces, the Ghana Police Service or those who have proof of holding a health insurance policy. Persons eligible to membership are expected to pay a contribution of GHC 7.2 per year equivalent of US$ 7.74 at time of passage of Act.

While the debate on VAT or not VAT was on going, labour took issue with the 2.5% of the Social Security and National Insurance Trust (SSNIT) to be appropriated to the insurance fund. Labour had earlier in 2002 agreed to the 2.5% as a matter of principle. Because the Trade Union Congress (TUC) representative served on the Ministerial Committee it was assumed TUC endorsed the proposal.

First, the TUC opined that as a matter of principle they had agreed to having a scheme that benefitted their members and to contribute in a way. However, that was policy. SSNIT money was workers money was the tune. Government should have in their opinion come back to the table to negotiate the details and not spring it out on them in a proposed law. Secondly, they put forward a crude calculation. Taking 2.5% from the SSNIT fund will actually amount to 14.7% of the SSNIT fund. The total amount also meant that each worker was contributing an approximate 3.6 Ghana cedis, an equivalent of US$ 3.87 in 2003. Workers considered that this was unfair and it will affect their pension benefits. The TUC decided to call on its membership to reject the proposal.

Fighting a two front war was never a good idea. The government chose to build coalitions through careful compromises. The NPP with advice from the technical experts within the Ministry of Health did three things. (i) It offered Labour guarantees that government was only borrowing the money and that no part of the funds appropriate will be transferred or accounted against any worker’s end of service benefit. (ii) All contributors to SSNIT and their dependants as well as all pensioners on SSNIT benefit will not have to pay premiums to be registered on the scheme. In effect, labour and for that matter the formal sector will have free health care in Ghana. (iii) Since formal sector labour has put up calculations on how much the 2.5% will be in terms of individual obligations, the informal sector will have to make up an equivalent in premiums. However, each informal sector contributor will have to carry one child so that all other dependants may benefit. Above 70 year olds were to be entirely exempted to be consistent with end of pension calculation for surviving pensioners which was pegged at 72 years. It was these negotiations that led to the setting of the premium at 7.2 Ghana cedis per annum or US$ 7.74.

Once these compromises were negotiated, a coalition was built between government and labour that led labour to tone down and not engage with the VAT issues. It was possible that without this coalition building government could not have passed the bill. Here, labour as civil society, proved more powerful an ally to have than to compromise with opposition political parties in parliament. Technically, it should be admitted that a better engagement process should have been developed with the key stakeholders by the technical experts to ameliorate some of these issues coming up late in the process.

## Discussions

Initiating a policy change requires thorough understanding of the context and careful management of the process. During the introduction of health insurance, politicians, technical experts, the media and scientific evidence played significant roles in setting the health insurance agenda. Politicians needed to understand the political gains and technical implications. These are then crystallize and re-positioned by them in the perceptive mind of the populace in such a way that gave them leverage with the electorate.

Various stakeholders had the opportunity to interact on common platforms and to express their views. In what resembles Dye’s bursts of ideas gaining credence over time think tanks such as the Institute of Economic Affairs (IEA) become a place of vibrant ideas that shifted depending on the audience. The institute suffered a few embarrassments in the process. For instance, the institute held a forum in June 2002 to discuss the ‘proposed Health Insurance Bill’ only to be told by the Deputy Minister of Health there was nothing so called. Yet, there had been several ministerial and government statements indicating that a bill had been drafted and under discussion. What the IEA tabled was actually a leaked draft which contained some of the difficult proposals that resulted in the polarisations discussed. Without adequate information and transparency on how the agenda is discussed and moved into policy and implementation, sometimes the entire enterprise can be viewed as conspiracy at work. This probably is what accounted for the political powerful that Agyepong and Adjei [5] observed in their article.

Constituency preservation proved to be important particularly for political parties when the agenda has already gained currency among the population. The way the issue is captured in their manifestos ultimately determines the acceptance of the proposition among their own constituents. Fundamentally, it became clear to us that political parties found it necessary to preserve who they are in order that their identity may be preserved against the elegy of foreign ideology. Clarity and clear messaging is important if the preferred option is to be supported by technical experts for implementation. This is what defined the difference between the success of the NDC who started insurance but without a clear orientation and ideology and the NPP who tied their political fortunes to the introduction of health insurance. To gain support for policy, the direction must be clear and uniquely defined as a product so that technical experts, civil society and the general public may engage without feeling politically drawn in.

Constituency preservation could also be through omission and it needs to be watched out. There was almost a general silence on the part of external partners as between 2000 and 2004. DANIDA was the only active partner in establishing community schemes and is credited with establishing some 34 community schemes. Though USAID commissioned reports into understanding factors contributing to sustainable management of community scheme, it did not actively express a position as an agency. The International Labour Organisation only engaged on requests to support actuarial analysis under the NDC.

As a collective body during the Health Summits, the development partners only advocated caution towards migrating too quickly to a national social health insurance scheme. Between 2001 and 2002 the aide memoirs signed show that health partners offered a very measured support for a community based insurance scheme with national oversight. In 2003 the partners requested that the bill be withdrawn because they disagreed with the design. Their preference was for community schemes. The Technical Committee rejected the request and partners threatened to march on parliament. After several discussions, it became clear that the differences were more of a misunderstanding in design and misinformation in the financing framework. The partners however refused to fund the scheme establishment directly.

One year after its passage, the World Bank Country Assessment Strategy (CAS) broke ranks and promised that the bank will provide a policy note that will aim to help the government to develop an implementation strategy for the recently-approved National Health Insurance Act, to ensure its fiscal sustainability and poverty impact. Because of their non-engagement from the start, the World Banks’ project to support the implementation of the scheme has failed. The Aide Memoire “Republic of Ghana Implementation Support Mission for Health Insurance Project (Credit-P101852) December 1 to 10, 2010” submitted by the Bank to government expressed disappoint in the lack of interest of government in external support for health insurance.

Clarity creates symbols and symbols define the constituent’s preferences. With high levels of clarity, the position of the dominant sponsoring organisation becomes linked more precisely with the policy proposal. It then becomes easy for opposing forces to engage through symbols manipulation. This is why VAT generated so much heat. However, the ambiguities and veneering of the same as representing something else also did not help. Although it will be hypothesis at this stage to say that coming clean that the 2.5% is actually an increase in VAT could have helped, it was still deceptive and surely did not help matters. It required too much energy on the part of the technical experts to try and find compromises through parliamentary lobbying. We should also admit that we did not have expertise in backroom lobbying and learnt it in the process. Being aware of the political environment is also very important. There were times when our recommendations have not been taken seriously, or have been set aside, because the political timing was not right or the evidence we brought up came at an inopportune time in terms of the politics around the evidence.

During coalition building, the government was forced to make several tactical concessions to build a winning coalition. The opposition were very focused on the single issue of VAT and constituency preservation that they did not engage fully with the entire proposition. Civil Society Organisations such as the Trade Union Congress (TUC) proved technically inefficient at representing their constituencies or the interest of the population. Given their capacity constraints in what health insurance actually meant under the proposed design they did not invest the effort needed to understand the policy issues. In several instances, they wanted to act on their own, remained in an unhelpful mode of ‘opposition’ to government and resist rather than engage with the policy processes. Although such contestation is justified in some instances, overall TUC lost the opening provided them to respond to the new opportunities that have arisen. It was therefore much easier for government to buy them out with concessions that directly appealed to their unionised members’ immediate interest while masking the actuarial mathematics of the outcome on return on investment of opportunities forgone. In the process, TUC failed to look at the big picture as representing all labour both SSNIT and non-SSNIT contributors such as the informal sector which contributed significantly to the Gross Domestic Product of the country. The NDC, the party in opposition attempted to call attention to this but did not have effective machinery in place to sell this interest. In our opinion, what was compromised was the general interest of the larger informal sector that had no organised voice at the table.

## Conclusion

The poor are still paying and the gainfully employed have free care. This is the dilemma of the realised policy and national health insurance in Ghana. Parliament no longer acted as the representative government protecting the interest of the people. It was polarised and caught up in party politics. Constituency preservation became more important than focus on the agenda and its benefits. Though the media attempted to articulate the issues, once the debate was on in parliament, it spent time on reporting routine government speeches, political party responses and was caught up in the sensationalism surrounding the feuds on insurance levy through VAT and SSNIT appropriations. The alternate explanation is that health insurance was a new ground and the media had little technical knowledge to analyse and report effectively. This capacity in the media needs to be build for new reforms that require new technical terminologies and design.

From the Ghana experience, evidence for policy is as essential as the political leverages used to interpret and arrive at desired outputs. It is sometimes better to analyse the policy and possible outcome by looking at the contextual factors and the epistemologies of the stakeholders rather than the stakeholders themselves. There are no straight and determined pathways for achieving outcomes so appreciating the evidence and basis for design, negotiation process and building coalitions along the way are skills to be mastered. Using the four-way policy and political leverages framework can help in the process. This framework is not meant to be used as an alternative to but as complementary to existing frameworks so an appreciation and deeper understanding of the subtle leverages is formed as part of policy formulation. In applying the framework, new perspectives have been brought to the introduction of health insurance as a policy in Ghana. We believe that this framework with others should bring a more balanced understanding to some of the policy and political leverages that come to play in introducing a major reform such as health insurance in the public sector.

## Competing Interests

The authors declare that they have no competing interests.

Author AS was a policy adviser and involved in the policy design, implementation and monitoring of the national health insurance scheme between 1997 and 2004. He was a member of the Ministerial Task Force on establishing the national health insurance scheme until March 2005. Since February 2011, he assumed the position of Coordinator of National Health Insurance Scheme in the Ministry of Health. Author SA was Director of the Policy Planning, Monitoring and Evaluation of the MOH between 2001 and 2004 and led the entire policy design, development of the legislation and negotiations of the Scheme. He was appointed the first Executive Secretary of the National Health Insurance Council in 2004 a position he held until 2007. He served in that capacity as a member of the insurance council.
